# Metagenomics Reveals the Microbial Community Responsible for Producing Biogenic Amines During Mustard [*Brassica juncea* (L.)] Fermentation

**DOI:** 10.3389/fmicb.2022.824644

**Published:** 2022-04-29

**Authors:** Yangyang Yu, Lu Li, Yujuan Xu, Hong Li, Yuanshan Yu, Zhenlin Xu

**Affiliations:** ^1^Guangdong Provincial Key Laboratory of Food Quality and Safety, College of Food Science, South China Agricultural University, Guangzhou, China; ^2^Sericultural & Agri-Food Research Institute, Guangdong Academy of Agricultural Sciences, Key Laboratory of Functional Foods, Ministry of Agriculture, Guangdong Key Laboratory of Agricultural Products Processing, Guangzhou, China; ^3^Institute of Agro-Products Processing, Yunnan Academy of Agricultural Sciences, Kunming, China

**Keywords:** biogenic amines (BAs), microbial community, metagenomic, fermentation, mustard

## Abstract

Biogenic amines (BAs) are considered potential hazards produced during fermented food processing, and the production of BAs is closely related to microbial metabolism. In this work, the changes of BA content were analyzed during mustard fermentation, and microbes and gene abundance responsible for producing BAs were revealed by metagenomic analyses. The results showed that cadaverine, putrescine, tyramine, and histamine were generated during mustard fermentation, which mainly accumulate in the first 6 days of fermentation. According to the metagenome sequencing, the predominant genus was *Bacillus* (64.78%), followed by *Lactobacillus* (11.67%), *Weissella* (8.88%), and *Leuconostoc* (1.71%) in the initial fermentation stage (second day), while *Lactobacillus* (76.03%) became the most dominant genus in the late stage. In addition, the gene abundance of BA production enzymes was the highest in the second day and decreased continuously as fermentation progressed. By tracking the source of the enzyme in the KEGG database, both *Bacillus* and *Delftia* closely correlated to the generation of putrescine. Besides, *Bacillus* also correlated to the generation of tyramine and spermidine, and *Delftia* also correlated to the generation of cadaverine and spermine. In the processes of fermentation, the pH of fermented mustard showed slower decrease compared with other similar fermented vegetables, which may allow *Bacillus* to grow at high levels before the pH <4. This study reveals the change of BA content and microbes involved in BA formation during mustard fermentation.

## Introduction

Fermentation is an important food processing and preservation method, which can be applied to milk, fish, meat, and vegetables. Fermentation of raw materials can not only extend shelf life but also generate unique and popular sensory characteristics, such as flavor and color (Torres et al., 2020). In addition, fermented products are reported to generate many health benefits such as anti-mutagenic, anti-carcinogenic, and anti-oxidative effects (Guan et al., 2021). However, despite such beneficial effects, reports indicate that undesirable metabolisms of several microbes can also generate toxic compounds during food fermentation, such as biogenic amines (BAs) (Feddern et al., 2019). BAs are harmful nitrogenous compounds with low molecular weight, which can be identified in various fermented foods, such as cheese, wines, and soy sauce (Ten Brink et al., 1990; Barbieri et al., 2019). Ingestion of foods containing high levels of BAs may lead to intoxication with symptoms such as headaches, nausea, diarrhea, flushes, and heart palpitations (Ruiz-Capillas and Herrero, 2019). In addition, BAs may be converted to potentially carcinogenic N-nitrosamines in the presence of nitrites (Shalaby, 1996; Shao et al., 2021). BAs form mainly *via* the microbial decarboxylation of amino acids (Park et al., 2019b). The typical BAs in fermented foods include tryptamine, putrescine, cadaverine, histamine, and tyramine, as a result of microbial decarboxylation of tryptophan, ornithine, lysine, histidine, and tyrosine, respectively (Li et al., 2019; Majcherczyk and Surowka, 2019).

Mustard [*Brassica juncea* (L.)], an important leafy green cruciferae vegetable, is rarely cooked as fresh vegetables because of its bitter and peppery taste. Generally, fresh mustard is processed with fermentation in salted brine, one of China’s most popular fermented vegetables. Briefly, fresh mustard is washed, drained, and immersed in 3–6% NaCl solution (*w*/*v*) in jars. And then, the jars containing mustard are sealed and stored an ambient temperature (20–25°C) for 10–15 days for maturation. Previous reports revealed that BAs appear during its fermentation, and in general, their concentration increases with time (Kosson and Elkner, 2010). It has been reported that the dominant BAs in fermented vegetables are mainly putrescine, tyramine, cadaverine, and histamine, and the range of BA contents in different fermented vegetables has also been reported (reports show the ranges of 23–32, 89–206, 10–32, 95–162, and 38–80 mg/kg for histamine, tyramine, tryptamine, putrescine, and cadaverine, respectively) (Sìwider et al., 2020). Multiple studies have proposed the maximum limits of BA content in food products: 100 mg/kg for histamine, 100–800 mg/kg for tyramine, and 1,000 mg/kg for total content of BAs (Ten Brink et al., 1990). Lee et al. (2019) reported that the contents of histamine and tyramine in fermented onions in Korea exceeded the proposed maximum limits by a factor of four and two, respectively. Thus, the presence of BAs in fermented foods (and non-fermented foods as well) has become one of the most important food safety issues.

Biogenic amines were significantly affected by complex microbial communities. Yu et al. (2021) investigated the correlations between microbial communities and BAs by 16S rRNA metagenomics, which showed that tryptamine positively correlates with *Lactobacillus* and *Pseudomonas*, while cadaverine is positively correlated with *Leuconostoc*. Nevertheless, the genes responsible for encoding the amino acid decarboxylase inside foods processed by fermentation were unable to be examined. Recently, metagenomic approaches allowed researchers to unveil the effect of complicated microbial communities inside fermented foods. Metagenomics can decipher the structure of the whole microbial community and identify the metabolic capability and gene outlines of such a system (Chen et al., 2017; Wu et al., 2017). However, the changes in genetic and biochemical mechanisms responsible for BA production during mustard fermentation have not been studied by metagenomic approaches. In this study, the changes of BA content were analyzed during mustard fermentation. Furthermore, microbes and gene abundance responsible for producing BAs were revealed by metagenomic analyses.

## Materials and Methods

### Sample Collection

One kilogram of fresh mustard was immersed in 2 L of cold boiled water with 3% (*w*/*v*) salt and fermented for 15 days at an ambient temperature (25°C) in a jar for anaerobic fermentation by naturally generating microbes from the materials. Nine duplicate jars of fermented mustard were made, and three of them were blended as one sample. The samples in different stages (1, 2, 4, 6, 9, 12, and 15 days) were collected to study the succession of physicochemical properties and BAs throughout the whole fermentation duration. Triplicate brine samples were collected on the 2nd, 6th, and 12th days as representative samples to study the framework and functions of microbial communities produced. The brine of the samples (10 ml) was centrifuged at 3,500 *g* for 10 min (4°C). Then, sediments were preserved by using liquid nitrogen and waited for subsequent DNA extraction.

### Physicochemical Properties During Mustard Fermentation

One gram of each sample was homogenized with 10 ml sterile distilled water for 30 min, and pH values were measured using a pH meter (PHS-3C, Shanghai, China). Total titratable acidity (TTA) (%) was determined by adopting titration of a product homogenate and using 0.1 mol l^–1^ NaOH according to the Association of Official Analytical Chemists [AOAC] (2002) procedures, and the content (mg/kg) of TTA is expressed as lactic acid.

The 3,5-dinitrosalicylic acid (DNS) method was adopted to determine the content of reducing sugar (Miller, 1959). One gram of a sample was combined with 9 ml of distilled water and then homogenized and centrifuged. Later, the supernatant (2 ml) was blended with 2 ml DNS. After boiling at 80°C for 10 min and cooling in ice water, the absorbance was measured at 550 nm using a UV-Vis spectrophotometer (UV-2550, Shimadzu). The content (mg/g) of reducing sugar is expressed as glucose equivalents.

Salinity was measured using Mohr’s titration (Chen et al., 2005). One gram of a sample was combined with 9 ml of distilled water and then homogenized and filtered. Ten milliliters of the filtered sample were mixed with 1 ml of 2% potassium chromate indicator and titrated with 0.02 M AgNO_3_ solution until the solution became reddish brown (the endpoint of the titration). The salinity was calculated using the formula below, in which *c* represents the concentration (mol/l) of the AgNO_3_ solution; 0.05844 represents the millimolar mass of NaCl; *m* represents the quality (g) of sample; *V*_0_ represents the volume consumed (ml) of AgNO_3_ solution for blank control; and *V*_1_ represents the volume consumed (ml) of AgNO_3_ solution for sample.


$$\text{Salinity}\left(\%\right)=\frac{c\times \left(V_{1}-V_{0}\right)\times 0.05844}{m}\times 100$$

### Measurement of Biogenic Amine Concentrations

The BA content was detected according to a previous report by Li et al. (2018). One gram of a sample was homogenized with 3 ml of 0.4 M HClO_4_ and extracted for 1 h. One gram of a sample was homogenized with 3 ml of 0.4 M HClO_4_ and extracted for 1 h. The mixed substance was centrifuged at 3,000 × *g* for 10 min, followed by the collection of the supernatant. Then, the supernatant (250 μl) was mixed with 25 μl of NaOH (2 M) and 75 μl of saturated NaHCO_3_ and reacted with 500 μl of dansyl chloride (5 mg/ml) at 55°C for 40 min. The reactant, after being blended with 25 μl of 25% NH_4_OH, was incubated at 55°C for 10 min. Then, the solution was filtered through a 0.22-μm membrane for high-performance liquid chromatography (HPLC) analysis. The HPLC analysis was conducted on an Agilent HPLC system (Agilent Ltd., Santa Clara, CA, United States) with an Eclipse XDB-C18 (4.6 mm × 250 mm, 5 μm) column. The elution solution was formed with acetonitrile (A) and water (B) using the gradient elution program as follows: 0–4 min, 50% A; 4–22 min, 50–90% A; 22–30 min, 90–50% A; 30–35 min, 50% A. The flow rate, injection volume, detection wavelength, and column temperature were specified as follows: 0.8 ml/min, 10 μl, 280 nm, and 35°C.

### Measurement of Free Amino Acids

Free amino acids (FAAs) were measured according to the protocols described by a previous study (Qi et al., 2021). Briefly, samples were extracted and purified by mixing them with 20% sulfosalicylic acid solution for 1 h. The mixed substance, after being filtered through a 0.22-μm filter and centrifuged, was measured by an amino acid analyzer (A300, MembraPure GmbH, Berlin, Germany).

### DNA Extraction, Library Construction, and Sequencing

Total genomic DNA was extracted from brine samples using the E.Z.N.A.^®^ Stool DNA Kit (D4015-02, Omega, Inc., United States) according to the manufacturer’s instructions. The metagenomic DNA libraries were generated with 2-μg genome DNA by applying the TruSeq™ DNA Sample Prep Kit (Illumina, San Diego, CA, United States). The average insert size is 350 bp. The quality of all libraries was assessed using an Agilent Bioanalyzer in combination with a DNA LabChip 1000 kit. Sequencing was conducted at LC-BIO TECHNOLOGIES CO., LTD. (Hangzhou, China) with an Illumina Genome Analyzer system pursuant to the manufacturer’s protocol. The sequencing data were submitted to NCBI Sequence Read Archive (SRA^1^) with accession number PRJNA780248.

### Bioinformatics Analysis

To obtain valid data for further analysis, subsequent raw reads were subject to the following process (Liu et al., 2019a): (1) removal of low-quality reads (length <50 bp, a quality value <20, or with ambiguous “N” bases); (2) removal of reads with over 10 ambiguous N bases; (3) removal of reads that shared an overlap of over 15 bases with the adapter. Trimmed reads were obtained, and they were *de novo* assembled to establish the metagenome for every sample by IDBA-UD v1.1.1. MetaGeneMark v3.26 was used to estimate all coding regions (CDS) of metagenomic contigs.

CD-HIT v4.6.1 clustered all samples’ CDS sequences to obtain gene catalog (unigenes) (Fu et al., 2012; Lee et al., 2021). The quantity of reads aligned by bowtie2 v2.2.0 was considered to forecast unigene abundance of a specific sample by transcripts per million. Alignment by using DIAMOND v 0.7.12 according to the NCBI NR database was made to acquire the lowest common ancestor taxonomy of unigenes. At the same time, functional annotation (KEGG) of unigenes was gained.

The abundance of unigenes were calculated using the formula below, in which *r* represents the number of reads mapped to the unigenes, and *L* represents the unigene length (Zeller et al., 2014).


$$G_{k}=\left(r_{k}/L_{k}\right)/\cdot \sum_{i}^{n}\left(r_{i}/L_{i}\right)$$

### Statistical Analysis

All the data were expressed as the mean ± standard deviation (SD) of triple measurements and subject to analysis of variance (ANOVA) testing with SPSS17.0 (SPSS, Inc., Chicago, IL, United States). Duncan’s test at *p* < 0.05 was adopted to compare multiple means. The figures of data were plotted by applying Origin v2021b (Origin Lab Corporation, United States).

## Results

### Changes in Physicochemical Properties

**FIGURE 1:**
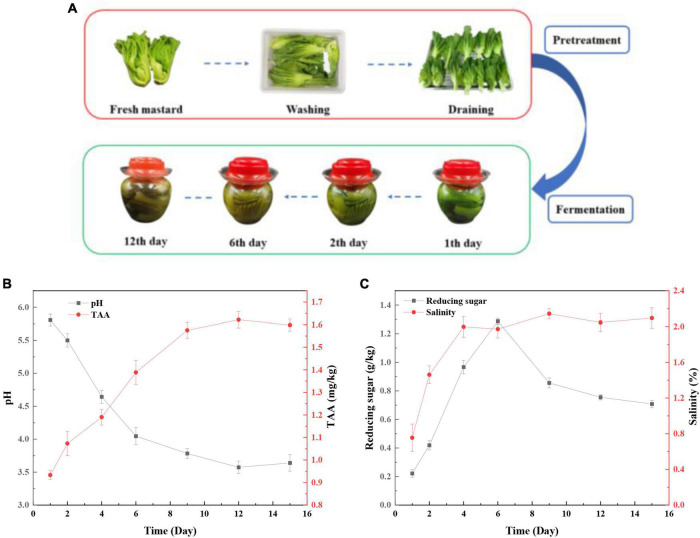
Changes in the physicochemical properties during the mustard fermentation. **(A)** The process of pretreatment and fermentation of fresh mustard. **(B)** pH and total titratable acidity (TTA). **(C)** Reducing sugar and salinity.

The changes in the appearance of mustard during fermentation are shown in Figure 1A. The raw mustard became yellow and softened gradually from the first day to the sixth day, and then, the color remained steady until the end of fermentation. The pH values decreased sharply and reached a low level of pH 3.78 in the first 9 days (Figure 1B). Then, the pH value was relatively stable during the following days. In contrast, TTA increased to 1.58 mg/kg during the first 9 days, and then remained stable above 1.5 mg/kg (Figure 1B). Generally, the fermented vegetables are considered to be ripe when the pH is below 4.0 (Zhang et al., 2016). This indicated that mustard had reached maturity after fermentation for 9 days. Reducing sugar is used as a carbon source in microorganisms and acts a key factor for growing the microorganisms (Jung et al., 2016). In addition, salinity increased to 3.5 g on the fourth day and then remained relatively stable until the end of fermentation (Figure 1C). However, the content of reducing sugar increased firstly and then decreased (Figure 1C). The consumption rate of sucrose reduced in the late fermentation period due to the metabolism of microbes, which was inhibited by the accumulation of lactic acid and a decrease of pH.

### The Change of Biogenic Amines and Biogenic Amine Precursors During Mustard Fermentation

**FIGURE 2:**
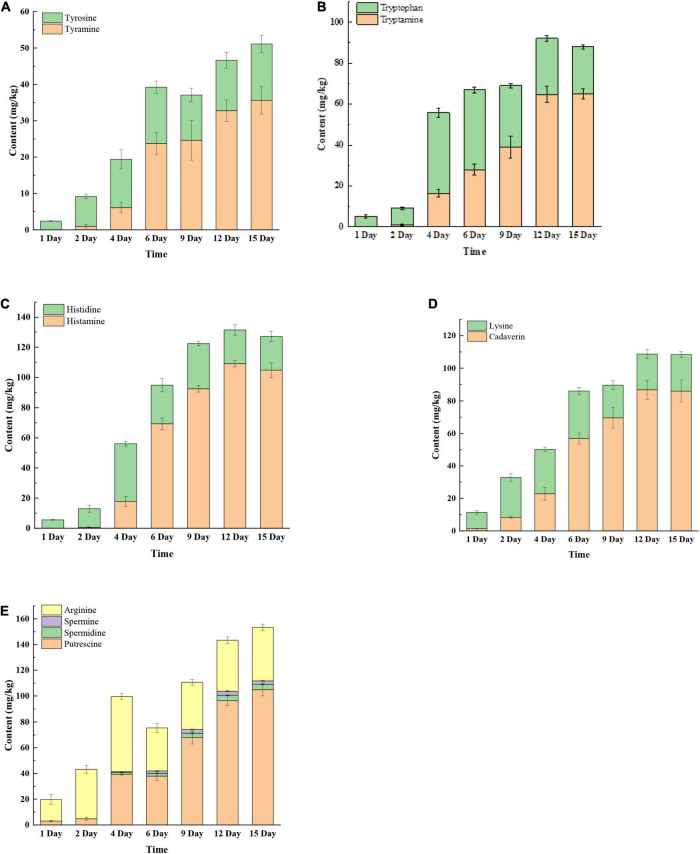
The changes of biogenic amines (BAs) and BA precursor amino acids at different fermentation times. **(A)** Tyrosine and tyramine; **(B)** tryptophan and tryptamine; **(C)** histidine and histamine; **(D)** lysine and cadaverine; **(E)** putrescine, spermidine, spermine, and arginine.

Biogenic amines are produced by decarboxylation of amino acids. As shown in Figure 2, seven BAs and their precursors (tyrosine, tryptophan, histidine, lysine, and arginine) were detected during mustard fermentation. Tyrosine, tryptophan, histidine, lysine, and arginine increased rapidly in early days of fermentation (fourth or sixth day). Subsequently, tyrosine, tryptophan, lysine, histidine, and arginine remained relatively stable until the end of fermentation. On day 1, there were only two BAs detected in these samples with very low concentrations, including cadaverine and putrescine (Figures 2D,E). After 2 days, tryptamine, tyramine, histamine, cadaverine, and putrescine accumulated rapidly then gradually plateaued (Figures 2A–E). After 9 or 12 days, putrescine and tyramine decreased slowly. In contrast, spermidine slowly increased until the end of fermentation (Figure 2E). At the late fermentation stage (12th or 15th day), the concentration of BAs and their precursors remained relatively stable. This may be due to changes in the pH, salinity, and microbial community affecting the expression of amino acid decarboxylase, which requires further verification.

The concentrations of BAs in the final products (at 15 days) were 35.62 mg/kg (tyramine), 65.00 mg/kg (tryptamine), 104.79 mg/kg (histamine), 86.02 mg/kg (cadaverin), 104.88 mg/kg (putrescine), 4.12 mg/kg (spermidine), and 2.77 mg/kg (spermine) (Figure 2). The result suggested that the dominant BAs were tyramine, tryptamine, histamine, cadaverine, and putrescine in fermented mustard. This was similar to the study of Sìwider et al. (2020), who reported that tyramine, tryptamine, histamine, cadaverine, and putrescine were the main BAs in traditional fermented vegetables.

### Overview of Metagenomic Data

A hierarchical cluster analysis (HCA) revealed that the mustard fermentation process could be divided into three groups based on physicochemical properties (pH, TTA, reducing sugar, and salinity) and BAs: group 1 (days 1, 2, and 4), group 2 (days 6 and 9), and group 3 (days 12 and 15) (Supplementary Figure 1). The principal component analysis (PCA) is shown in Supplementary Figure 1, indicating that there were differences in the physicochemical properties and BA content of samples on the 2nd, 6th, and 12th day. Therefore, in order to better understand the mustard fermentation process, samples on days 2, 6, and 12 were selected for the following metagenomic assay.

**FIGURE 3:**
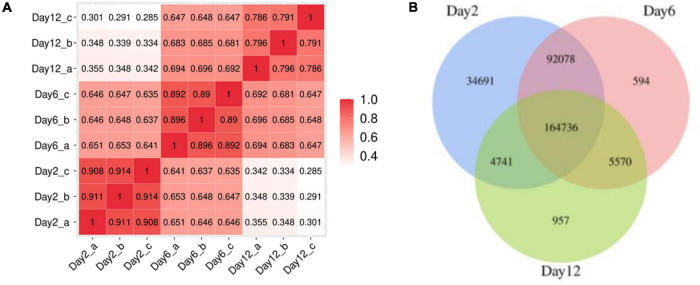
**(A)** Correlation analysis of the number of predicted gene among the samples; **(B)** Venn diagram of the distribution of unigenes in fermentation broth samples.

A total of 837,234,160 raw reads from nine samples (average of 93,026,018 reads per sample) were generated. After quality control, a total of 577,754,078 clean reads (average of 48,146,173 reads per sample) were thus generated. In total, >97% of the reads had sequencing errors of <1% (Q20, Supplementary Table 1), showing the high quality of the sequencing procedure. After predicting ORFs by MetaGeneMark, 303,367 ORFs were identified inside the nine samples with 43.44% of these genes possessing a complete ORF (Supplementary Table 2). The correlation analysis of the number of predicted gene showed a difference between extra-groups, while it showed a good correlation between intra-groups (Figure 3A). It suggested that the samples have a good repeatability. The number of unigenes decreased from 296,255 at the 2nd day to 176,004 at the 12th day (Figure 3B), and the largest unique unigenes (34,691) were found in samples of the 2nd day. This result suggested that a large shift occurred in the gene constitution during the mustard fermentation.

### Microbial Community Composition During Mustard Fermentation

**FIGURE 4:**
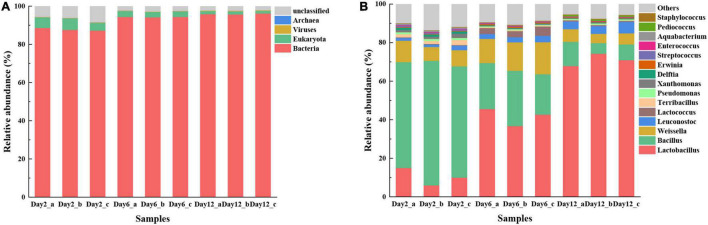
Microbial community composition present in fermented mustard at different fermentation stages based on metagenomic analyses. Composition is shown at kingdom **(A)** and genus levels **(B)**. Only those taxonomic groups with >0.02% relative abundances are shown.

Metagenomic sequences were compared against the NCBI NR database to identify the microbial communities in different fermentation stages. According to Figure 4A, the relative abundance of bacteria was the highest throughout the whole fermentation process, while the abundance of *Eukaryota*, *Viruses*, and *Archaea* was much lower. The abundance of bacteria increased from 87.82 to 95.91%, while the relative abundance of *Eukaryota* gradually decreased over time (5.21 to 1.50%). The result is similar to a previous report that *Bacteria* might be the dominant microbes during the fermentation of potherb mustard (Liu et al., 2021).

At the genus level, 89.64% of the unigenes could be annotated, while only 53.24% of the unigenes could be annotated at the species level, so the description at the genus level was more scientific for descripting the microbial characterization. The top 15 genera are shown in Figure 4B. The predominant genus was *Bacillus* (59.11%), followed by *Weissella* (8.87%), *Lactobacillus* (10.33%), and *Leuconostoc* (1.65%) in the initial fermentation stage (second day). This is different from previous studies on Sichuan Paocai by Xiao et al. (2018b). The results of the latter studies showed that *Pediococcus*, *Streptococcus*, *Acinetobacter*, *Staphylococcus*, *Serratia*, and *Brevibacterium* were present as major populations in an early fermentation period (0–2 days). Different raw materials may cause such differences because microbial communities in an early fermentation period at the initial fermentation stage mainly originated from raw materials (Xiao et al., 2020). The relative abundance of *Bacillus*, *Terribacillus*, and *Xanthomonas* reduced rapidly as the fermentation progressed, while the relative abundances of *Lactobacillus* generally increased. In addition, the relative abundance of *Weissella* rose until the 6th day, and subsequently, it declined fast at the 12th day. The above results clearly disclosed remarkable differences in the microbial abundance, diversity, and composition during mustard fermentation.

### The Source of Enzymes and Unigene Abundance in the Metabolic Pathway of Biogenic Amines

**FIGURE 5:**
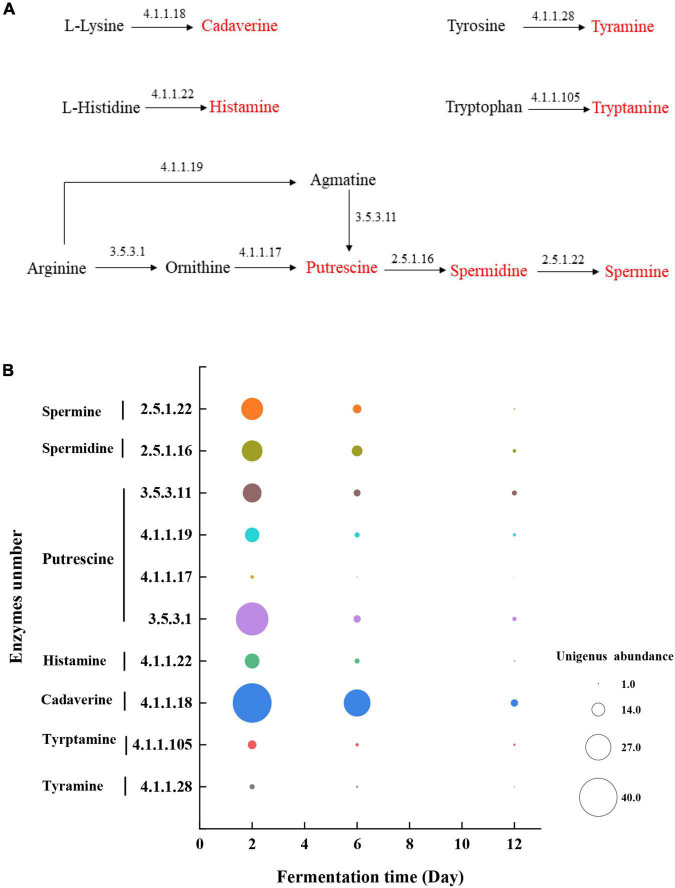
**(A)** Pathway of production of BAs and **(B)** the unigene abundance of the annotated enzymes in microbial community during mustard fermentation.

As shown in Figure 5A, tyrosine, tryptophan, lysine, and histidine are respectively activated by a one-step decarboxylation reaction to generate tyramine, tryptamine, cadaverine, and histamine. However, the production of putrescine depends on the ornithine and agmatine produced by arginine reaction as the precursor substances. By tracking the source of the enzyme in the KEGG database, tyrosine decarboxylase (4.1.1.28) mainly came from *Bacillus* and *Terribacillus*, which can convert tyrosine to tyramine (Table 1). Tryptophan decarboxylase (4.1.1.105) mainly came from *Weissella* and *Deinococcus*, which can convert tryptophan to tryptamine (Table 1). L-lysine decarboxylase (4.1.1.18) could convert L-lysine to cadaverine, which mainly came from *Delftia*, *Weissella*, *Lactobacillus*, and *Aquabacterium*. In addition, *Weissella* and *Aquabacterium* also produce L-histidine (4.1.1.22) decarboxylase, which can convert covert L-histidine to histamine (Table 1). However, putrescine is formed from arginine *via* ornithine decarboxylase (EC:3.5.3.1; EC:4.1.1.17) or agmatine deaminase (EC:4.1.1.19; EC:3.5.3.11) pathways, and these key enzymes were mainly from *Bacillus* and *Delftia* (Table 1). Furthermore, putrescine is a precursor of spermidine, which is produced by the spermidine synthase (EC:2.5.1.16) produced by *Bacillus* and *Terribacillus* and spermidine produced by the spermidine synthase (EC:2.5.1.22) produced by *Delftia* and *Aquabacterium* (Table 1).

**TABLE 1:** The source statistics of enzymes in metabolic pathways of biogenic amines.

Reaction pathway	EC no.	Source of genes involved in enzyme synthesis (genus)
Tyrosine → tyramine	4.1.1.28	*Bacillus* *Terribacillus*
Tryptophan → tryptamine	4.1.1.105	*Weissella* *Deinococcus*
L-lysine → cadaverine	4.1.1.18	*Delftia* *Weissella* *Lactobacillus* *Aquabacterium*
L-histidine → histamine	4.1.1.22	*Weissella* *Aquabacterium*
Arginine → ornithine → putrescine	3.5.3.1; 4.1.1.17	*Bacillus* *Delftia* *Aquabacterium*
Agmatine → agmatine → putrescine	4.1.1.19; 3.5.3.11	*Bacillus* *Delftia*
Putrescine → spermidine	2.5.1.16	*Bacillus* *Terribacillus*
Spermidine → spermine	2.5.1.22	*Delftia* *Aquabacterium*

Figure 5B shows the unigene abundance of the annotated enzymes during mustard fermentation. As fermentation progressed, the unigene abundance of the annotated enzymes decreased continuously. After 12 days of fermentation, the unigene abundance of the annotated enzymes almost disappeared. This affected the expression of enzymes, which resulted in a high concentration of BA precursor substances without transforming BAs at the late fermentation stage.

## Discussion

pH and TTA are basic properties during fermentation as they could affect the growth of the microorganisms and the accumulation of metabolic products, which can affect the flavors of fermented vegetables (Wu et al., 2015). Therefore, pH is used to assess the fermentation stage of fermented vegetables. In this study, the pH of 4 was only reached after 8 days of fermentation, which showed slower pH decrease compared with other similar fermented vegetables. For example, the pH was <4 after 3 days in German sauerkraut (Muller et al., 2018), and the pH was <3.7 after 3 days in homemade Chinese sauerkraut (Yang et al., 2020). There are differences in pH reduction time, which may be caused by fermentation conditions (i.e., temperature and salinity). It was previously reported that either increasing the temperature (Wang et al., 2020) or decreasing salinity (Lee et al., 2021) in a certain range is beneficial for rapid pH decrease and shortening of the fermentation time. In addition, the slow decrease in pH during mustard fermentation may have led to microbial growth associated with BA accumulation, and this needs to be investigated further.

Fermented mustard is a traditional product of vegetable fermentation that is produced by spontaneous fermentation with microorganisms coming from raw materials (Yu et al., 2021). During fermentation, BAs are generally produced by microorganisms through enzymatic decarboxylation of amino acids (Park et al., 2019a). The excessive consumption of BAs is associated with adverse toxicological effects. However, the content of BAs in fermented vegetables has no official limitations. Histamine is the only BA that is officially limited in fish products, and it is regulated to be below 50 mg/kg by the U.S. Food and Drug Administration [FDA] (2011) and below 100 mg/kg by the EFSA Panel on Biological Hazards (2011). In this study, histamine content was above 100 mg/kg on the 12th and 15th day of fermentation, which exceeded the limit level suggested by the FDA (50 mg/kg) and EFSA (100 mg/kg). In addition, after fermentation, fermented mustard also contains a high concentration of tyramine, tryptamine, cadaverine, and putrescine. Despite the unobvious toxicity of these BAs, they can react with nitrite to generate carcinogenic nitrosamine (Xiao et al., 2018a; Sun et al., 2019). In addition, the presence of BAs is an effective indicator of food spoilage since microbial food spoilage may be accompanied by a rise in decarboxylase production (Hao and Sun, 2020). Therefore, the study and control of microbes facilitating the generation of BAs during food fermentation have drawn great attention. Fermented food microbiota is directly associated with the content of BAs in food fermentation. Therefore, it is important to monitor the potential microbial producers of BAs in fermented foods and to manage their population. Previous studies using the 16S rRNA approach analyzed the changes of microbial community in Sichuan suancai during fermentation, and the results indicated that environmental microorganisms, such as *Micrococcaceae*, from raw materials dominated at the initial stage of fermentation, followed by *Leuconostocaceae*, and then *Lactobacillaceae* at the late stage of fermentation (Xiao et al., 2018b). The present study shows that at the genus level, the relative abundance of *Bacillus*, *Terribacillus*, and *Xanthomonas* reduced rapidly as the fermentation progressed, while the relative abundances of *Lactobacillus* generally increased and became the most dominant genus in a late stage. This result was similar to a previous study that *Lactobacillus* generally increased and fermented to produce acid during kimchi fermentation, which could inhibit *Bacillus* (Lee et al., 2021). *Lactobacillus* became the most dominant genus in the late stage, and its relative abundance increased to 71%. This is consistent with previous studies on fermented cabbages (Liang et al., 2020), fermented mustard (Liu et al., 2021), and other fermented vegetables (Liu et al., 2019b). The great abundance of *Lactobacillus* during the final phase of mustard fermentation maturation could be due to their tolerance to a wide range of environmental conditions (i.e., low pH and high salt concentrations). In this study, the pH was approximately 4.0 and TTA reached 1.5 mg/kg after fermentation for 8 days. It has been reported that *Lactobacillus marxianus* can produce fatty acids, organic acids, alcohols, and other flavor substances through fermentation (Torres et al., 2020). In addition, Zheng et al. (2020) proposed reclassification of the genus *Lactobacillus* into 25 genera based on whole-genome sequences. Because the old database was used in metagenomic analyses in this study, *Lactobacillus* in the paper does not only refer to the few host-adapted species of the *Lactobacillus delbrueckii* group and *Paralactobacillus* according to the new taxonomy but also possibly to other genera created from this reclassification.

The capability of BA production by the genera (*Lactobacillus*, *Bacillus*, and *Delftia*) has been demonstrated in sufu (Hu et al., 2021) and cheese (Tittarelli et al., 2018). Nevertheless, because environmental factors affect gene expression (Xie et al., 2020), the capability of bacteria to produce BAs in fermented mustard needs to be verified by isolation and BA-generation assays. In general, *Bacillus* and *Delftia* were important genera of BA production in fermented mustard (Table 1). *Bacillus* is associated with the production of tyramine, putrescine, and spermidine, while *Delftia* is correlated with the production of cadaverine, putrescine, and spermine. These results indicated that diverse microbes like *Bacillus* and *Delftia* might facilitate the generation of BAs during the fermentation process. Therefore, controlling the growth of *Bacillus* and *Delftia* during the initial stage of fermentation will benefit to reduce the concentration of BAs. In this study, we analyzed the abundance of genes and microbes associated with biosynthesis of BAs, which provided theoretical support for controlling the BAs in mustard fermentation processes. Nevertheless, it is necessary to isolate the representative strains from the dominant communities to evaluate their capabilities to produce BAs, which will be more accurate to elucidate the metabolite-formation mechanisms in microbial of fermentation mustard. In this study, we investigated the microbial contribution to BAs during mustard fermentation using metagenomics and showed that diverse bacterial species harbored genes associated with the production of various BAs. Furthermore, the effects of different fermentation, processing, and storage conditions, in addition to storage times, on BA production and BA-producing bacterial species must be further investigated.

## Data Availability Statement

The datasets presented in this study can be found in online repositories. The names of the repository/repositories and accession number(s) can be found in the article/Supplementary Material.

## Author Contributions

YaY and LL collected the data and wrote the manuscript. YX analyzed the data. HL revised the manuscript. YuY and ZX designed the experiments and reviewed the manuscript. All authors contributed to the article and approved the submitted version.

## Conflict of Interest

The authors declare that the research was conducted in the absence of any commercial or financial relationships that could be construed as a potential conflict of interest.

## Publisher’s Note

All claims expressed in this article are solely those of the authors and do not necessarily represent those of their affiliated organizations, or those of the publisher, the editors and the reviewers. Any product that may be evaluated in this article, or claim that may be made by its manufacturer, is not guaranteed or endorsed by the publisher.

## Footnotes

1
http://www.ncbi.nlm.nih.gov/Traces/sra
