# Supplementary material for: Metagenomics Reveals the Microbial Community Responsible for Producing Biogenic Amines During Mustard [Brassica juncea (L.)] Fermentation
Source: Front Microbiol. 2022 Apr 29;13:824644. doi: 10.3389/fmicb.2022.824644 (PMC9100585; doi:10.3389/fmicb.2022.824644)
Supplement: Supplementary file 1 [file Data_Sheet_1.docx]

**Title:**Metagenomics reveals the production mechanism of biogenic amine in suancai during the process of mustard fermentation

**Running Title:** Microbes of producing biogenic amines

**Author:** YangyangYu^a,b^, Lu Li^b^, YujuanXu^b^, Hong Li^c^, YuanshanYu^b,^ * andZhenlinXu^a,^*

^a^ Guangdong Provincial Key Laboratory of Food Quality and Safety, College of Food Science, South China Agricultural University, Guangzhou 510642, China

^b^ Sericultural & Agri-Food Research Institute, Guangdong Academy of Agricultural Sciences/Key Laboratory of Functional Foods, Ministry of Agriculture/Guangdong Key Laboratory of Agricultural Products Processing, Guangzhou, 510610 China

^c^Institute of Agro-Products Processing, Yunnan Academy of Agricultural Sciences, Kunming 650032, China

*** Corresponding author:** Mailing address: No. 133, DongGuanZhuang RD., TianHe District, Guangzhou, P. R. China, 510610

**Phone:** +86-15975596649

**E-mail address:** yuyuanshan2016@qq.com (Yuanshan Yu), jallent@163.com (Zhenlin Xu

**Figure S1.**HCA analysis based on physicochemical properties (pH, TAA, Reducing sugar and salinity)and BAsin different fermentation times.

**Figure S2.**PCA analysis of physicochemical properties andBAsinsamples on 2^th^, 6^th^ and 12^th^ day.

**Table S1.** Overview of the read statistics of the metagenomic sequence data set.

| Sample | Raw Data  Read | | Clean Data  Read | | Clean% | | Q20% | | Q30% | | GC% | |
| --- | --- | --- | --- | --- | --- | --- | --- | --- | --- | --- | --- | --- |
| Day2_a | 90857400 | | 87268346 | | 96.05 | | 97.90 | | 93.89 | | 40.36 | |
| Day2_b | 95335718 | | 93065526 | | 97.62 | | 97.64 | | 93.46 | | 40.12 | |
| Day2_c | 94058786 | | 91551598 | | 97.33 | | 97.85 | | 93.87 | | 40.23 | |
| Day6_a | 99711544 | | 91776452 | | 92.04 | | 98.55 | | 95.25 | | 43.11 | |
| Day6_b | 92842764 | | 86344028 | | 93.00 | | 98.54 | | 95.25 | | 42.88 | |
| Day6_c | 80768562 | | 74525926 | | 92.27 | | 97.96 | | 93.73 | | 43.07 | |
| Day12_a | 93125972 | | 83328322 | | 89.48 | | 98.62 | | 95.48 | | 44.36 | |
| Day12_b | 93881712 | | 84185456 | | 89.67 | | 98.66 | | 95.60 | | 44.82 | |
| Day12_c | 96651702 | 88367598 | | 91.43 | | 98.56 | | 95.32 | | 44.88 | |  |

**Table S2.** Statistical information of gene catalogue of fermented mustard

| ORFs | 303367 |
| --- | --- |
| integrity:start^a^ | 68678(22.64%) |
| integrity:end^b^ | 69587(22.94%) |
| integrity:all^c^ | 131779(43.44%) |
| integrity:none^d^ | 33323(10.98%) |
| Total Len.(Mbp) | 163.13 |
| Average Len.(bp) | 537.74 |
| GC percent | 43.82% |

^a^gene has initiation and termination codon

^b^ gene only has initiation codon

^c^ gene only has termination codon

^d^ gene has no initiation and termination codon
